# Beginning to See the Light: Lessons Learned From the Development of the Circadian System for Optimizing Light Conditions in the Neonatal Intensive Care Unit

**DOI:** 10.3389/fnins.2021.634034

**Published:** 2021-03-18

**Authors:** Esther M. Hazelhoff, Jeroen Dudink, Johanna H. Meijer, Laura Kervezee

**Affiliations:** ^1^Laboratory for Neurophysiology, Department of Cellular and Chemical Biology, Leiden University Medical Center, Leiden, Netherlands; ^2^Department of Neonatology, Wilhelmina Children’s Hospital and Brain Centre Rudolf Magnus, University Medical Centre Utrecht, Utrecht, Netherlands

**Keywords:** cycled light, development, preterm infants, circadian system, Neonatal Intensive Care Unit, chronobiology, eye development and function

## Abstract

The circadian timing system optimizes health by temporally coordinating behavior and physiology. During mammalian gestation, fetal circadian rhythms are synchronized by the daily fluctuations in maternal body temperature, hormones and nutrients. Circadian disruption during pregnancy is associated with negative effects on developmental outcomes in the offspring, highlighting the importance of regular and robust 24-h rhythms over gestation. In the case of preterm birth (before 37 weeks of gestation), maternal cues no longer synchronize the neonate’s circadian system, which may adversely affect the neonate. There is increasing evidence that introducing robust light-dark cycles in the Neonatal Intensive Care Unit has beneficial effects on clinical outcomes in preterm infants, such as weight gain and hospitalization time, compared to infants exposed to constant light or constant near-darkness. However, the biological basis for these effects and the relationship with the functional and anatomical development of the circadian system is not fully understood. In this review, we provide a concise overview of the effects of light-dark cycles on clinical outcomes of preterm neonates in the NICU and its alignment with the development of the circadian system.

## Introduction

During pregnancy, a highly controlled uterine environment provides the best possible conditions for optimal fetal development in preparation for a successful transition to postnatal life. The mother supplies oxygen, hormones, and nutrients via the placenta. In addition to these substrates, the mother conveys circadian timing cues to the fetus through her own daily rhythms in body temperature, physical activity, feeding behavior, and hormonal levels (Serón-Ferré et al., 2001, 2012; Bates and Herzog, 2020). Several lines of evidence show that maternal circadian rhythms are important for the offspring. For example, pregnant women on shift work, which may lead to circadian disruption, have an increased risk of adverse reproductive outcomes (Kervezee et al., 2018). A recent meta-analysis reported an increased odds of preterm birth (odds ratio [OR]: 1.13; 95% confidence interval [CI]: 1.00–1.28) and of having a small-for-gestational-age neonate (OR: 1.18; 95% CI: 1.01–1.38) in rotating night shift work compared to fixed day shift work (Cai et al., 2019). Research in animal models supports these findings, showing that environmental disruption of circadian rhythms during pregnancy leads to adverse pregnancy outcomes (Summa et al., 2012) as well as to negative effects in the offspring later in life, such as altered adrenal function, impaired adaptive immune system, social avoidance and depressive like behavior (Borniger et al., 2014; Cissé et al., 2017a,b; Man et al., 2017; Smarr et al., 2017; Salazar et al., 2018).

In case of preterm birth (i.e., babies born before 37 weeks of gestation), the period that the fetus spends in the controlled intrauterine environment comes to an end prematurely. The preterm baby is typically admitted to a Neonatal Intensive Care Unit (NICU), where clear environmental 24-h rhythms are usually absent, exposing the infant to a constant temperature and irregular or continuous illumination (Fielder and Moseley, 2000; White et al., 2013; Bueno and Menna-Barreto, 2016; Hay, 2017). There is a growing concern that the absence of temporal cues in the NICU may not be optimal for the development of premature infants (Symington and Pinelli, 2006; van den Hoogen et al., 2017). The postnatal environment is especially important given the critical period of development that occurs *ex utero* following extreme preterm birth (i.e., born before 28 weeks of gestation). For example, between 18 and 39 weeks of gestation, fetal brain volume usually increases 10 to 34-fold, depending on the brain region (Andescavage et al., 2017; Matthews et al., 2018). In addition, during the stay in the NICU, the immature and rapidly developing brain is particularly vulnerable to environmental stressors, which might compromise neurobehavioral development (Aucott et al., 2002; Ward and Beachy, 2003; Symington and Pinelli, 2006; Santos et al., 2016; Matthews et al., 2018; Twilhaar et al., 2018). Indeed, as the survival rate of premature infants has improved (Zeitlin et al., 2010; World Health Organization, 2012; Platt, 2014), we have to acknowledge that the potential complications of prematurity, both experienced in the NICU and in later life, become increasingly important (Ward and Beachy, 2003). Designing a NICU environment from a chronobiological perspective might be desirable for improving preterm outcomes.

There is accumulating evidence that introducing a robust light-dark cycle in the NICU environment is beneficial for postnatal development (as reviewed below). However, to better understand the biological basis for these effects, it is important to take into consideration the different critical periods of fetal development. For the light-dark cycle to be beneficial for postnatal development, a premature infant should be able to perceive light and confer this light information to the biological clock. In this review we aim to provide an overview of the potential effects of a light-dark cycle in a NICU in the context of the development of the visual and circadian systems.

## The Circadian Timing System

Over the course of evolution organisms have adapted to alternating light-dark cycles due to the rotation of the earth on its axis by developing a circadian clock, which controls the daily timing of biological processes ranging from gene expression to behavior (Dibner et al., 2010). In mammals, circadian rhythms are orchestrated by a central clock located in the suprachiasmatic nuclei (SCN) of the hypothalamus (Sollars and Pickard, 2015). The SCN network autonomously generates a circadian rhythm and transmits this temporal information to peripheral oscillators that are present in virtually all other cell types in the body. The SCN is synchronized to the external environment by light input that is transmitted from the retina directly to the SCN through a neural pathway called the retinal hypothalamic tract (RHT) (Hughes et al., 2015).

Within the retina, light is perceived by a subgroup of retinal ganglion cells that express the photopigment melanopsin, which renders them intrinsically photosensitive. Modulated by input from rods and cones (Van Diepen et al., 2013; Barrionuevo et al., 2014; Spitschan et al., 2014), these intrinsically photosensitive retinal ganglion cells (ipRGCs) transmit non-visual light information not only to the SCN, but also to other brain areas involved in non-image forming functions such as the olivary pretectal nucleus, which is involved in the regulation of the pupillary light reflex (Chen et al., 2011; La Morgia et al., 2018).

## Circadian Entrainment by Light-Dark Cycles in the Neonatal Intensive Care Unit

Introducing a robust light-dark cycle in the NICU has been suggested as a strategy to entrain the circadian system in preterm infants, which may support growth and development as well as help prevent complications frequently experienced by the preterm infant such as disturbances in body temperature, sleep or feeding patterns (Morag and Ohlsson, 2016). Currently, lighting conditions in NICUs across the world are highly variable, owing to flexible guidelines (e.g., see Best et al., 2018). For example, the Consensus Committee for NICU Design, recommends illuminance levels to be between 10 and 600 lux in the NICU (White et al., 2013). NICUs can be distinguished by observing either constant light or constant near-darkness, with light levels below 20 lux throughout the 24-h period (Morag and Ohlsson, 2016).

Several studies have evaluated the effect of introducing a robust light-dark cycle (‘cycled light’) compared to either constant light or near-darkness on clinical outcomes in preterm infants in NICUs, such as weight gain. A systematic review by the Cochrane library concluded that cycled light seems to shorten the length of hospital stay, although the evidence is hampered by the small sample size and the inability to blind the intervention (Morag and Ohlsson, 2016). Cycled light may also improve other outcomes, such as weight gain and the incidence or retinopathy of prematurity (Morag and Ohlsson, 2016). Specifically, the clearest benefits were found in studies that compared exposure to cycled light to exposure to constant light conditions in NICUs. Several studies reported that cycled light compared to continuous light was associated with improved weight gain (Mann et al., 1986; Miller et al., 1995; Vásquez-Ruiz et al., 2014; Farahani et al., 2018), although the magnitude of the effect is difficult to compare between the studies. For example, Farahani et al. (2018) found that infants in cycled light conditions gained significantly more weight between day 9 after birth and discharge from the NICU than infants in constant conditions (29 ± 7 g per day vs 15 ± 6 g, per day; mean ± SD), while Mann et al. (1986) found no difference in weight at discharge, but 3 months after discharge, infants that had been exposed to cycled light in the NICU were on average 500 g heavier than those exposed to continuous light. Furthermore, one study found that the length of the hospitalization period was significantly shorter in infants exposed to cycled light condition compared to those exposed to continuous light (34 ± 3 days vs 51 ± 5 days; mean ± SEM) (Vásquez-Ruiz et al., 2014). Another study found an effect of similar magnitude on hospitalization period (59 ± 28 days in the cycled light group vs 75 ± 25 days in the continuous light group), although this difference was not statistically significant (Miller et al., 1995). However, it should be noted that other studies did not find an effect of cycled light on hospitalization duration (Mann et al., 1986; Farahani et al., 2018; Moselhi Mater et al., 2019). Cycled light interventions in the NICU have also been reported to increase nighttime sleep duration (Guyer et al., 2015) and the ratio of daytime to nighttime activity (Blackburn and Patteson, 1991; Watanabe et al., 2013), to reduce or stabilize heart rate patterns (Blackburn and Patteson, 1991; Vásquez-Ruiz et al., 2014), and lower distress levels compared to continuous light (Moselhi Mater et al., 2019). Interestingly, one study reported a difference in morning and evening melatonin levels in infants exposed to cycled light but not in infants exposed to continuous light (Vásquez-Ruiz et al., 2014), suggesting that implementing a light-dark cycle promotes circadian entrainment. Although the long-term benefits of improved circadian entrainment in infants early in life remain to be investigated, improved circadian entrainment, provided that it leads to more robust sleep-wake cycles, would be of immediate benefit to the parents after the infant’s release from the NICU.

Other studies compared outcomes in preterm infants exposed to cycled light compared to constant near-darkness. One study found that preterm infants receiving cycled light at birth or from gestational week 32 onward gained significantly more weight than those exposed to constant near-darkness until gestational week 36 (Brandon et al., 2002). However, most studies reported no significant effects on body weight at discharge or body weight gain (Boo et al., 2002; Rivkees et al., 2004; Guyer et al., 2012) and length of hospital stay (Brandon et al., 2002, 2017; Rivkees et al., 2004; Guyer et al., 2012). One underpowered study reported a clinically relevant, but not statistically significant effect of initiating a cycled light intervention at a gestational age of 28 weeks compared to cycled light started at 36 weeks on weight gain (mean weight gain of 193.8 g vs 176.3 g between gestational week 36 and 44) (Brandon et al., 2017). In addition, while a cycled light intervention did not affect the emergence of day/night differences in body temperature compared to constant near-darkness in preterm infants (Mirmiran et al., 2003), the development of distinct rest-activity cycles was accelerated in preterm infants exposed to cycled light compared to those exposed to constant near-darkness (Rivkees et al., 2004), although this effect was not observed in another study (Lebel et al., 2017). Finally, exposure to cycled light in the NICU reduced fussing and crying per 24 h after discharge to home compared to exposure to constant near-darkness (Guyer et al., 2012). Of note, the observation that some effects of cycled light interventions are only observed after discharge from the hospital highlight that long-term follow-up is essential to better understand the effects of cycled light in the NICU (Mann et al., 1986; Rivkees et al., 2004).

**FIGURE 1:**
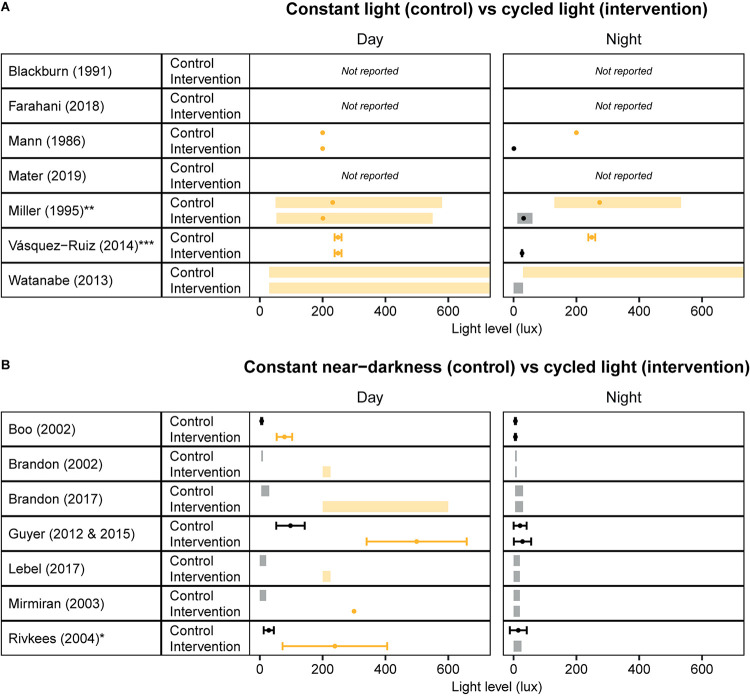
Overview of light levels used in cycled light studies that have been conducted in Neonatal Intensive Care Units. Light levels reported in studies that investigated the effect of cycled light versus **(A)** constant light and **(B)** constant near-darkness. Values are depicted as ranges (shaded rectangles), means (dots), and/or standard deviations (error bars), depending on the values reported in the publication. Yellow/Orange colors indicate “light”, Black/Gray colors indicate “darkness”. *Standard errors reported in Rivkees et al. (2004) are converted to standard deviations by multiplying with the square root of the n per group. **Light intensities measured in the horizontal plane are reported here. ***Unclear whether standard deviations or standard errors are reported; using published values for error bars.

Altogether, the available literature provides a strong rationale to further evaluate strategies to improve circadian entrainment in NICUs. However, a more systematic approach is needed that considers the underlying physiological mechanisms by which cycled light may exert its beneficial effects. An important point of consideration is that light levels varied from study to study, and are often not precisely reported – if at all (Figure 1). This makes it hard to compare different studies and make recommendations regarding the optimal light levels. Therefore, future research is warranted to determine what lighting regimes, in terms of intensity and spectral composition, and at what developmental time point, lead to optimal clinical outcomes.

## Development of the Circadian System, From Light Input to Functional Output

**FIGURE 2:**
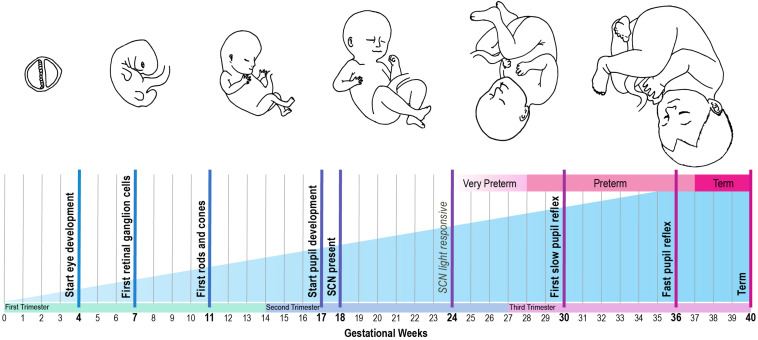
Developmental timeline of the human circadian system, based on reviewed human and animal studies. Bold text: developmental time points that are obtained from human studies. Italic text: developmental time points that are inferred from animal studies, using the gestational age equivalent to humans.

The use of cycled light as a strategy to promote circadian entrainment and improve clinical outcome should be considered in context of the marked developmental changes of the visual and circadian system that occur in preterm infants during their stay in the NICU. At different stages of gestation, the different anatomical structures of the visual and circadian systems develop (Figure 2). This starts on gestational day 17, when the first structures of the eye are formed (Van Cruchten et al., 2017). In infants born at term (between gestational week 37–41), the structural development of the eye takes place nearly completely *in utero*, although the further functional maturation is not completed until at least 4 years after birth (Hendrickson and Yuodelis, 1984).

The pupil can be viewed as the anatomical gateway that modulates the transmission of light from the environment to the circadian system. Its development starts around gestational week 17. However, its function in regulating the amount of light that reaches the retina develops only later in gestation. From 30 weeks onward preterm infants start to show slow alterations in pupil diameter, and from 34 weeks gestation they have a clear and fast pupil reflex to changes in illumination (Robinson and Fielder, 1990). However, as the babies are still developing, there might be some differences in thickness of the eyelids, which may affect the amount of light entering the eye. Robinson et al. (1991) showed that a higher percentage of red light penetrates the eyelid in prematurely born babies compared to adults, but how this changes over the course of development is unknown.

Secondly, the effect of light on circadian entrainment is influenced by the function of the different retinal cells. The retinal ganglion cells are formed from gestational week 7 onward, well-before the emergence of rods and cones between week 10 and 12 (Van Cruchten et al., 2017). Expression of melanopsin is present in human eye tissue at least by gestational week 8.6 (Tarttelin et al., 2003). Melanopsin may even be present earlier, since this was the earliest developmental timepoint that was included in the study by Tarttelin et al. (2003). For instance, in mice, melanopsin is expressed by embryonic day 10.5–11.5 in mice (equivalent to gestational week 4 in humans) (Tarttelin et al., 2003). This coincides with the emergence of retinal ganglion cells in mice, which occurs at an earlier developmental stage compared to humans (Tarttelin et al., 2003). Of note, while rod and cone photoreceptors only become functional two weeks after birth in mice, melanopsin-expressing retinal ganglion cells are light responsive immediately at birth (Sekaran et al., 2005), and possibly even earlier during gestation (Rao et al., 2013). This light response is mediated by melanopsin and, strikingly, was found to have a functional role for vascular patterning *in utero*: a higher degree of retinal vascular overgrowth was observed in pups reared in constant darkness during late gestation compared to those reared in a light-dark cycle (Rao et al., 2013). This effect seems to be mediated by melanopsin, because a null-mutation in the melanopsin gene *Opn4* caused the same phenotype (Rao et al., 2013). These results suggest that light exposure *in utero* is already important for fetal eye development in mice. To what extent this applies to human fetal development remains to be investigated. However, these findings support the need to develop a clear mechanistic understanding of the effect of light-dark cycles on the development of the visual system in preterm infants at different developmental stages.

The ability of a neonate’s circadian system to entrain to the environmental light-dark cycle also depends on the functionality of the RHT. To the best of our knowledge no studies are available on the development of the human RHT. However, it is known that the SCN is light responsive in baboons born at term and can be entrained to a light-dark cycle with a light intensity of 200 lux (Rivkees et al., 1997). Furthermore, the SCN in preterm baboons responds to light from a stage equivalent to 24 weeks of gestation in humans onward (Hao and Rivkees, 1999). As the fetal development of primates and human is similar (Stouffer and Woodruff, 2017), these results suggest the projections from the retina to the SCN via the RHT may be functional from 24 weeks of gestation onward in human development.

For entrainment of the neonate to be possible, the environmental light input must be processed by a functional SCN. At what stage the SCN develops during gestation in humans is largely unknown. However, it has been shown that the SCN is present at 18 weeks of gestation, using melatonin receptor- and D1 dopamine receptor labeling (Reppert et al., 1988; Rivkees and Lachowicz, 1997; Rivkees, 2007). In addition, studies in squirrel monkeys support these findings, with SCN neurogenesis during early gestation (Reppert and Schwartz, 1984). At the end of gestation, a clear day-night difference in SCN glucose utilization can be observed in these monkeys (Reppert and Schwartz, 1984). Since only 20% of the total cell number found in adulthood is present at term (Swaab, 1995), further maturation of the human SCN takes place after birth.

Once the individual components of the circadian system are formed, they generate an output in the form an approximately 24-h rhythm that is projected to other brain areas and peripheral organs, which regulate circadian rhythms both in behavior and hormone production. In term babies, 24 h sleep-wake rhythms are not yet apparent directly after birth, but these typically emerge between 7 and 16 weeks of age (Jenni et al., 2006). Around the same time, day-night differences can also be observed in hormone production, such as melatonin from 12 weeks of age (Kennaway et al., 1992). In addition to rhythms in hormone production, heart rate shows a clear difference between day and night from 7 weeks onward (Hoppenbrouwers et al., 2012), whereas day/night differences in body temperature only become apparent from 2 months of age (Petersen and Wailoo, 1994). Whether the developmental timeline of these rhythms is influenced by the postnatal environmental light-dark cycle in preterm infants is unclear, as reviewed above.

Together, these studies show that the initial structures of the eye and central clock are present at 24 weeks of gestation, and that further maturation of both the eye and the SCN takes place largely in the third trimester of pregnancy and in the first few years after birth. With most of the structures formed by 24 weeks of gestation, it is conceivable that in the case of preterm birth, environmental light input is conveyed by the eyes to an SCN that is – at least partly – functional. The physiological mechanisms underlying the seemingly beneficial effects of cycled light in the NICU, such as weight gain and length of hospital stay, remain to be elucidated, although it has been suggested that reduced (nighttime) activity and lower, more stable heart rate rhythms, leading to reduced energy expenditure, may play a role (Miller et al., 1995).

## Discussion

As exposure to a bright day and dark night seems to positively influence weight gain, duration of hospital stay, and other clinically relevant measures, cycled light appears to be beneficial for the development of preterm babies. As the structures of the circadian system appear to be formed at 24 weeks, it is conceivable that the positive effects of cycled light are mediated through these structures. This fits with the striking observation that environmental light not only reaches the uterus in both sheep and mice (Parraguez et al., 1998; Rao et al., 2013) but also plays a role in retinal vascularization in the mouse fetus (Rao et al., 2013). To what extent light reaches the uterus in pregnant women, and whether light plays a functional role in developmental processes *in utero* in humans as well, are important questions that warrant further investigation.

In addition to the beneficial effects of cycled light, another topic that requires attention in future studies is the potential functional role of other environmental timing cues on circadian entrainment and clinical outcomes in the NICU, such as temperature and feeding cues. For example, the unborn fetus is exposed to fluctuations in maternal core body temperature *in utero*, which – in non-pregnant women – shows a circadian rhythm with an amplitude of around 0.4–0.5°C (Baker et al., 2001). Whether this rhythm persists during human pregnancy is unknown (Mark et al., 2017). Likewise, 24-h variations in the levels of circulating hormones, such as melatonin, may promote circadian entrainment of the unborn fetus (McCarthy et al., 2019). Furthermore, as a result of alternating periods of eating and fasting of the mother during pregnancy, the unborn fetus will be exposed to 24-h variations in nutrients, metabolites, and metabolic hormones, which may also function as timing cues for the circadian system (Wehrens et al., 2017; Lewis et al., 2020). In the NICU, where neonates typically receive intermittent or continuous feeding, this rhythmicity is largely lost. Therefore, optimizing 24 h rhythms in the environment – in the broadest sense – may be an opportunity to further improve a preterm infant’s health and development.

Much remains unknown regarding the mechanisms underlying the effect of environmental light/dark cycles on clinical outcomes in preterm infants. Based on the developmental timeline of the visual and circadian system, it seems that prematurely born infants, even those born extremely preterm, are functionally capable to sense light to some degree, since most of the photoreceptors and opsins are present during early gestation. With the formation of both the RHT and the SCN during the second trimester, the circadian system is at least partially functional from gestational week 24 onward.

Taken together, as the survival rate of preterm infants continues to rise, it becomes increasingly important to focus on optimizing the NICU environment and thereby help improve preterm infants’ health and wellbeing later in life. Several studies show beneficial effects of cycled light over constant light conditions, and a systematic review on the topic is cautiously positive (Morag and Ohlsson, 2016). For these findings to be implemented, larger studies are necessary, preferably with a long term follow-up and more precise reporting of experimental light conditions, such as the spectral composition of light. In this context, the minimal reporting guidelines for studies involving lighting interventions recently developed by Spitschan et al. (2019) may be helpful. In parallel, fundamental research should focus on providing a mechanistic understanding of these findings. Furthermore, the effect of other rhythmic timing cues such as temperature and feeding on clinical outcomes in preterm infants are a promising avenue to investigate in future studies.

## Author Contributions

EH and LK wrote and revised the manuscript. JD and JM revised the manuscript. All authors contributed to the article and approved the submitted version.

## Conflict of Interest

The authors declare that the research was conducted in the absence of any commercial or financial relationships that could be construed as a potential conflict of interest.
